# Mechanism of Guanosine Triphosphate Hydrolysis by the Visual Proteins Arl3-RP2: Free Energy Reaction Profiles Computed with Ab Initio Type QM/MM Potentials

**DOI:** 10.3390/molecules26133998

**Published:** 2021-06-30

**Authors:** Maria G. Khrenova, Egor S. Bulavko, Fedor D. Mulashkin, Alexander V. Nemukhin

**Affiliations:** 1 Chemistry Department, M.V. Lomonosov Moscow State University, Leninskie Gory 1/3, 119991 Moscow, Russia; mkhrenova@lcc.chem.msu.ru (M.G.K.); fedor.mulashkin@gmail.com (F.D.M.); 2 Bach Institute of Biochemistry, Federal Research Centre “Fundamentals of Biotechnology” of the Russian Academy of Sciences, 119071 Moscow, Russia; 3 Biology Department, M.V. Lomonosov Moscow State University, Leninskie Gory 1/3, 119991 Moscow, Russia; gangstarfull@gmail.com; 4 N.M. Emanuel Institute of Biochemical Physics, Russian Academy of Sciences, Kosygina 4, 119334 Moscow, Russia

**Keywords:** GTP hydrolysis, Arl3-RP2, reaction mechanism, Gibbs energy profiles, QM/MM, QM/MM molecular dynamics, biochemistry of vision

## Abstract

We report the results of calculations of the Gibbs energy profiles of the guanosine triphosphate (GTP) hydrolysis by the Arl3-RP2 protein complex using molecular dynamics (MD) simulations with ab initio type QM/MM potentials. The chemical reaction of GTP hydrolysis to guanosine diphosphate (GDP) and inorganic phosphate (Pi) is catalyzed by GTPases, the enzymes, which are responsible for signal transduction in live cells. A small GTPase Arl3, catalyzing the GTP → GDP reaction in complex with the activating protein RP2, constitute an essential part of the human vision cycle. To simulate the reaction mechanism, a model system is constructed by motifs of the crystal structure of the Arl3-RP2 complexed with a substrate analog. After selection of reaction coordinates, energy profiles for elementary steps along the reaction pathway GTP + H_2_O → GDP + Pi are computed using the umbrella sampling and umbrella integration procedures. QM/MM MD calculations are carried out, interfacing the molecular dynamics program NAMD and the quantum chemistry program TeraChem. Ab initio type QM(DFT)/MM potentials are computed with atom-centered basis sets 6-31G** and two hybrid functionals (PBE0-D3 and ωB97x-D3) of the density functional theory, describing a large QM subsystem. Results of these simulations of the reaction mechanism are compared to those obtained with QM/MM calculations on the potential energy surface using a similar description of the QM part. We find that both approaches, QM/MM and QM/MM MD, support the mechanism of GTP hydrolysis by GTPases, according to which the catalytic glutamine side chain (Gln71, in this system) actively participates in the reaction. Both approaches distinguish two parts of the reaction: the cleavage of the phosphorus-oxygen bond in GTP coupled with the formation of Pi, and the enzyme regeneration. Newly performed QM/MM MD simulations confirmed the profile predicted in the QM/MM minimum energy calculations, called here the pathway-I, and corrected its relief at the first elementary step from the enzyme–substrate complex. The QM/MM MD simulations also revealed another mechanism at the part of enzyme regeneration leading to pathway-II. Pathway-II is more consistent with the experimental kinetic data of the wild-type complex Arl3-RP2, whereas pathway-I explains the role of the mutation Glu138Gly in RP2 slowing down the hydrolysis rate.

## 1. Introduction

Calculations of energy profiles of chemical reactions in proteins constitute an important part of studies on enzyme catalysis. The application of different versions of the quantum mechanics/molecular mechanics (QM/MM) method is a commonly accepted practice in this field [1,2,3,4,5]. In many cases, essential features of catalytic reaction mechanisms can be revealed by the results of QM/MM calculations of minimum energy profiles on the potential energy surface of a model system, for example, [6,7,8]. Free energy reaction profiles provide additional important data. These profiles are estimated either by adding corresponding corrections to the potential energy stationary points or by using algorithms based on molecular dynamics (MD) calculations. In the latter case, reliable potentials should be applied in simulations, which account for chemical transformations in enzyme active sites. The use of QM/MM potentials in the MD algorithms is an attractive option; however, it is important to maintain a high accuracy level of quantum chemical methods in such QM/MM MD applications. Recent advances in interfacing [9] the MD package NAMD [10,11] with the quantum chemical package TeraChem [12]—which is highly efficient on GPUs—promise new achievements in modeling enzyme-catalyzed reactions. It is feasible to apply atom-centered basis sets of 6-31G** or cc-pvdz quality and to use a variety of hybrid functionals of the density functional theory (DFT) in calculations of energies and forces in QM parts and commonly accepted force field parameters in MM parts. When computing MD trajectories of about 10 ps length per window, it is possible to apply the umbrella sampling (US) scan of the Gibbs energy surface with the subsequent statistical analysis, using umbrella integration (UI) methods [13,14] to quantify elementary steps of chemical reactions in the enzyme active sites, for example, [15,16,17].

In this work, we apply the QM/MM MD method to compute the Gibbs energy profiles of one of the most important biochemical reactions, namely, the hydrolysis of guanosine triphosphate (GTP) to guanosine diphosphate (GDP) and inorganic phosphate (Pi), catalyzed by GTP-binding proteins, GTPases. These enzymes are responsible for signal transduction in living cells, and their malfunction due to point mutations may lead to the development of severe human diseases. Specifically, we select the protein complex Arl3-RP2 as an important example of GTP to GDP transforming machinery [18]. Arl3 is a member of the adenosine diphosphate-ribosylation factor-like (Arf-like or Arl) GTPases of the Ras superfamily. In the complex with the protein RP2 (retinitis pigmentosa 2), Arl3 mediates the traffic of components between the inner and outer segments of the eye photoreceptor cells [19,20].

According to experimental studies [21,22], the RP2 partner of the complex performs in the hydrolysis reaction as a GTPase-activating protein (GAP). Similar to other GTPases, the intrinsic hydrolysis rate of Alr3 is very slow (1.2·10^−5^ s^−1^), but upon binding to RP2, the reaction rate increases dramatically to 1.2 s^−1^. Several amino acid residues from RP2 are important for efficient hydrolysis. The RP2-Arg118 is a so-called “arginine finger” and its mutation to alanine decreases k_cat_ to approximately the same amount as in the intrinsic hydrolysis by Arl3 [21]. The value of arginine fingers in the enzyme catalysis is well recognized; see, for example, [23,24,25]. The role of another RP2 residue, Glu138, is also underlined in [21]; its mutation to the alanine results in a 100-fold decrease of k_cat_ in comparison with the wild type Arl3-RP2 complex.

**Figure 1:**
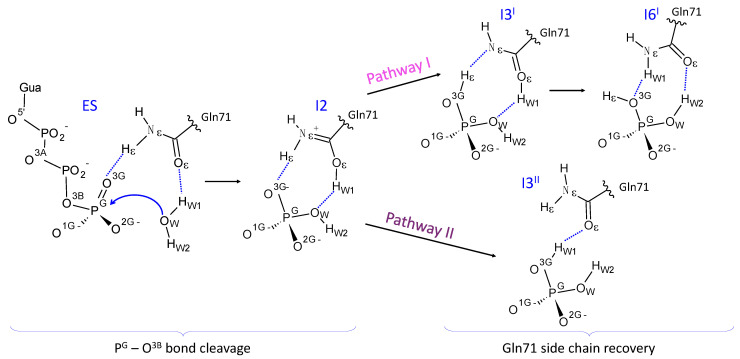
GTP hydrolysis by Arl3-RP2 proposed in [26] (Pathway I) and in this work (Pathway II).

Earlier [26], the potential energy profile of the GTP + H_2_O → GDP + Pi reaction in the Arl3-RP2 active site was evaluated using the QM(PBE0-D3/cc-pvdz)/MM(AMBER) method. The proposed mechanism is composed of six elementary steps. The first three of them describe the cleavage of the P–O bond in GTP and the formation of the inorganic phosphate, H_2_PO_4_^−^. The subsequent steps are required to restore the enzyme. This scenario corresponds to the so-called glutamine-assisted mechanism of GTP hydrolysis by GTPases [27,28,29,30,31], according to which the catalytically important glutamine residue (Gln71, in the case of Arl3) actively participates in the reaction being a part of the proton transfer chain. We outline in Figure 1 the basic features of the reaction scheme according to [26]. The reaction intermediates are called I1-I6 as in [26]. In the present paper, we add superscripts in Roman capitals (I or II) to some intermediate names for the reasons disclosed below. The Gln71 side chain in the reactant and product structures occurs in the conventional amide conformation; whereas the unstable I2 and I3^I^ intermediates include the Gln71 side chain in non-conventional forms. The I3^I^ intermediate includes the Gln71 side chain in the imidic acid form. In the I2 minimum energy point, the side chain of Gln71 is in the protonated form. The analytic fit of the kinetic curve corresponding to the enzyme-product accumulation leads to the effective rate constant, k_cat_, 0.015 s^−1^ [26], which is two orders lower than the experimental value of 1.2 s^−1^ [21].

The motivation of the present study is twofold. First, our aim is to compare the results of calculations of the Gibbs energy profiles using the QM/MM MD approach and results of calculations of the minimum energy profiles with QM/MM, which are performed with a similar QM-MM partitioning, a similar description of the QM subsystem, and are carried out by the same research team. Second, we intend to extend the knowledge on the structure and dynamics of the Arl3-RP2 machinery, which is an essential part of the human visual system. More precisely, we present an alternative pathway of the enzyme regeneration that better agrees with the experimental k_cat_ values for the wild-type Arl3-RP2 complex and for the mutant in which RP2-Glu138 is replaced by glycine. It should be noted that the details of the mechanism of GTP to GDP transformation in GTPases are still under debate [30,31,32], and this particular molecular system presents an important model for simulations of GTP hydrolysis.

## 2. Models and Methods

**Figure 2:**
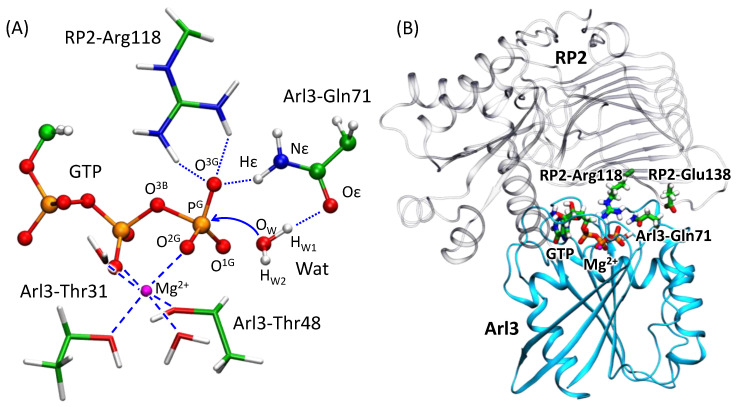
(**A**) Active site of the Arl3-RP2 complex in the ES conformation; (**B**) The Arl3-RP2 complex. Arl3 is shown in light blue cartoon representation and RP2 in transparent grey. Catalytically important residues, RP2-Arg118, RP2-Glu138 and Arl3-Gln71, GTP are shown in sticks. Here and in the following figures, blue dotted lines show hydrogen bonds and dashed lines—coordination bonds; atom color code: magnesium—magenta, oxygen—red, phosphorus—orange, nitrogen—blue, hydrogen—white and carbon—green.

In the reactive state of GTPase-GAP complexes, the GTP molecule and the nucleophilic water molecule are tightly bound in the protein cleft. We illustrate the corresponding enzyme–substrate (ES) complex in Arl3-RP2 in Figure 2, showing a part of molecular groups in the active site. In particular, the phosphate groups of GTP are coordinated by the magnesium ion Mg^2+^. The magnesium six-fold coordination shell also includes two structural water molecules and the hydroxyl groups of Thr31 and Thr48 from Arl3. A crucially important “arginine finger”, Arg118 from RP2, interacts with the phosphate groups of GTP in the cleft. The nucleophile water molecule, Wat, is oriented by the oxygen atoms of the carbonyl group of the Thr48 backbone and from the amide group of the Gln71 side chain, both from the Arl3. The side chain of Arg118 forms hydrogen bonds with the O^3G^ of the γ-phosphate group. Importantly, the RP2-Glu138 side chain is located apart from the active site in the ES complex and it is not evident how its mutation affects the observed [21] catalytic activity.

These structural features can be recognized in the crystal structure PDB ID 3BH7 of the Arl3-RP2-GDP-AlF_4_^−^ complex [21], which is used here as a source of coordinates of heavy atoms. To construct molecular models, the substrate (GTP) was manually restored. The hydrogen atoms were added by assuming positive charges of the side chains Lys, Arg and negative charges of Glu and Asp; the protonation state of His was manually suggested for every residue according to its local environment. The protein complex was solvated in the rectangular box of water molecules; the distance from the protein surface to the border of the cell was greater than 12 Å. These systems were neutralized by adding sodium ions. All-atom force fields were utilized; CHARMM36 [33,34] for the protein, GTP and magnesium cation and TIP3P [35] for the water molecules. Preliminary equilibration with classical force fields was performed for 5 ns at T = 300 K and p = 1 atm with a 1 fs integration time step. During this simulation, the harmonic potentials with the K = 40 kcal/mol/Å^2^ were added to the hydrogen and coordination bonds and were centered at 1.8 Å and 2.2 Å, respectively. The distance of the nucleophilic attack, d(P^G^…O_W_), was constrained by a harmonic potential with K = 80 kcal/mol/Å^2^ and centered at 2.9 Å. All classical MD simulations were performed with the NAMD program [10]. The cutoff distance for all non-covalent interactions was set to 12 Å with the switch to the smoothing potential at 10 Å. Equilibration of the system was checked by the RMSD value calculated over all non-hydrogen atoms. Last frames from the MD run were utilized for the subsequent preparation of the QM/MM models.

QM/MM MD simulations were performed using NAMD [10] and TeraChem [12] packages interfaced according to [9]. The QM part comprised a large fraction of the enzyme active site: the phosphate groups of GTP, the catalytic water molecule, the Arl3 side chains of Gly69, Gly70, Gln71, Thr48, Thr31 and Lys30, the backbones of Asp26 and Asn27, the RP2 side chain of Arg118, the magnesium ion Mg^2+^ and two water molecules from its coordination shell. A part of the calculations was performed by adding the RP2-Glu138 side chain to the QM subsystem. We performed two sets of calculations, both with the 6-31G** basis set [36,37] and two different functionals—PBE0 [38] and ωB97x [39]. In both cases, the dispersion correction D3 [40] was added. The first 1 ps of each MD trajectory was excluded from the analysis. The umbrella integration and the weighted histogram analysis [13,14] were used to construct the profiles. We prepared a set of files with atomic coordinates in the PDB format corresponding to the values of reaction coordinates close to those obtained for the minima and transition states in the umbrella integration analysis of the reaction coordinate distributions (see Results section and Data Availability Statement). 

The Markov state model (MSM) technique [41] was applied to simulate transitions between unstable reaction intermediates I1 ⇆ I2 ⇆ I3^I^ (see Figure 1). A set of QM/MM MD trajectories of the total length of 35 ps was executed for the MSM analysis using the PyEMMA 2 software [42]. The distances between the Hε and Nε atoms in Gln71 and between the Oε atom of Gln71 and H_W1_ from Wat served as the corresponding coordinates. The snapshots were split into 50 clusters via the k-center algorithm. Microstates were clustered into three macrostates, corresponding to I1, I2 and I3^I^. The choice of discretization was validated with the Chapman–Kolmogorov test.

## 3. Results

### 3.1. Dynamics of the Enzyme–Substrate Complex

Figure 1 illustrates an arrangement of molecular groups in the active site of the Arl3-RP2-GTP-H_2_O structure in the vicinity of the ES complex. Previous experience in theoretical studies of enzyme-catalyzed reactions [15,16,17] shows that dynamics of ES is an essential issue, which helps us to recognize reactive conformations of the system. Therefore, we analyze dynamical properties of ES complexes along MD trajectories calculated with the QM/MM potentials, also paying attention to the choice of the DFT functional in QM. As explained in the Methods section, the QM subsystem is treated at the Kohn–Sham DFT level using either PBE0-D3/6-31G** or ωB97x-D3/6-31G** approaches.

**Figure 3:**
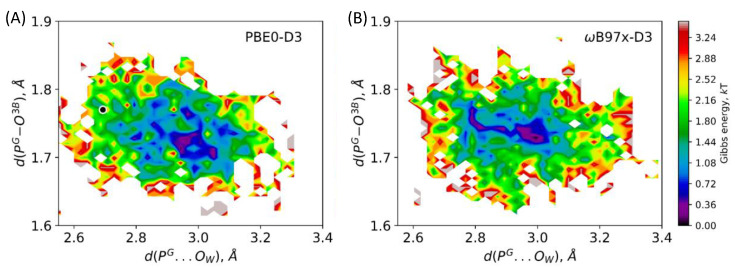
Maps demonstrating dynamic features of ES complexes in respect of two interatomic distances, d(P^G^–O^3B^) and d(P^G^…O_W_), calculated with the PBE0-D3 (**A**) and ωB97x-D3 (**B**) functionals in the QM/MM MD simulations. The color bar is in the *k*T units.

**Figure 4:**
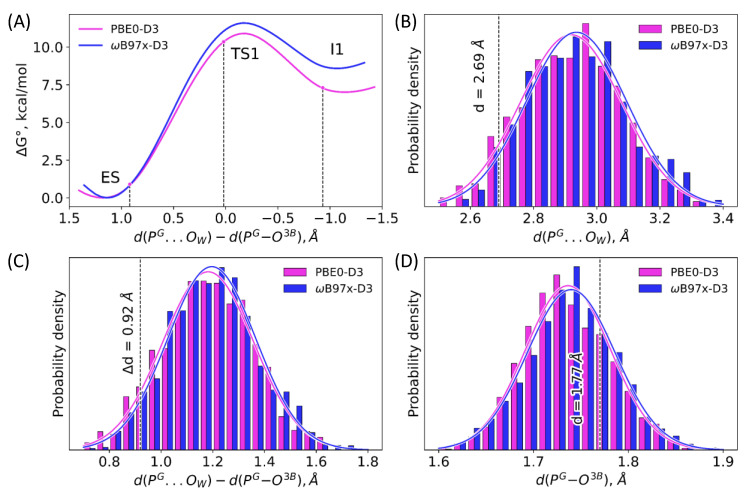
(**A**) Gibbs energy profiles of the first reaction step obtained in QM/MM MD simulations. (**B**–**D**) Distributions of the d(P^G^–O^3B^), d(P^G^…O_W_) and d(P^G^…O_W_)–d(P^G^–O^3B^) distances in the unconstrained QM/MM MD simulations. Black dashed lines correspond to the reaction coordinate values (**A**,**C**) and individual interatomic distances (**B**,**D**) extracted from the stationary points on PES [26]. Magenta and blue curves correspond to the calculations obtained with the PBE0-D3 and ωB97x-D3 functionals, respectively.

Figure 3 depicts distributions of two interatomic distances, d(P^G^–O^3B^) and d(P^G^…O_W_), calculated along the 10 ps unconstrained QM/MM MD trajectories of the ES complex obtained with two selected functionals. These geometry parameters, that is, the elongation of the covalent P^G^–O^3B^ bond to be cleaved and the distance of the nucleophilic attack, d(P^G^…O_W_), are responsible for the substrate activation. Both maps demonstrate similar features showing the minimum at d(P^G^–O^3B^) = 1.70 ÷ 1.75 Å and d(P^G^…O_W_) = 2.9 ÷ 3.1 Å. A slight distinction between the results of two functionals is seen in panels (A) and (B): there is a narrow low-energy pathway (dark blue) to the region with higher d(P^G^–O^3B^) and lower d(P^G^…O_W_) values if the system is treated with the ωB97x-D3 functional. This issue may be attributed to different compositions of the functionals. The ωB97x-D3 is a long-range corrected hybrid functional with the 100% exact HF exchange term in the long-range part that increases the ionic contribution and finally results in higher substrate activation, which is reflected by the elongation of the covalent P^G^–O^3B^ bond and the shortening of the distance of the nucleophilic attack. Similar features of both MD trajectories are also visualized in distributions of two considered distances (Figure 4, panels B, D). The covalent P^G^–O^3B^ bond distributions are 1.74 ± 0.05 Å for both calculation protocols. Similarly, the P^G^–O_W_ distance is 2.92 ± 0.16 Å and 2.94 ± 0.16 Å for ES complexes calculated at the PBE0-D3 and ωB97x-D3 levels, respectively. In Figure 3A, a white-circled black point shows the equilibrium geometry parameters of the ES complex obtained in QM/MM minimization [26]. This point lies quite apart from the minimum on the Gibbs energy surface and it is ~2 *k*T higher in energy. The P^G^–O^3B^ bond length is 1.77 Å, and it is elongated as compared with the corresponding values in the most populated area. The distance of the nucleophilic attack is 2.69 Å, that is, ~0.2 Å smaller than that on the Gibbs energy surface minimum. Therefore, we conclude that the ES geometry obtained on the potential energy surface, PES, corresponds to a tighter ES complex that should facilitate the nucleophilic attack.

### 3.2. Comparison of Reaction Energy Profiles for the Pathway Revealed in QM/MM Minimization

**Figure 5:**
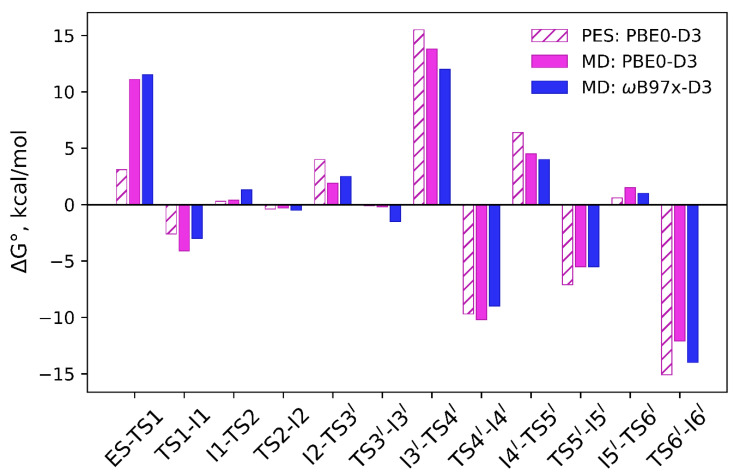
Gibbs energy differences between the neighboring minima and transition states. Pink bars correspond to the Gibbs energies obtained from the PES scan [26] with the subsequent addition of zero-point energies and entropic and thermal contributions, according to the statistical thermodynamic formulae. Magenta and blue bars correspond to the values obtained in this study using the umbrella sampling/umbrella integration method at the Kohn Sham PBE0-D3 and ωB97x-D3 levels, respectively.

In this subsection, we compare the results of free-energy calculations of the GTP hydrolysis by Arl3-RP2 obtained using various approaches. First, the Gibbs energy profile is evaluated by adding statistical thermodynamics correction terms to the potential energies of the stationary points [26]; the corresponding points are recalculated on the basis of the data presented in [26]. Second, the profiles are obtained with QM/MM MD using umbrella sampling simulations with subsequent statistical analysis of the reaction coordinate distributions. This comparison is important for estimating the influence of the conformational space sampling for a proper description of the reaction energy paths of enzymatic reactions. We also analyze here the role of the functional in QM. We focus on the energy differences between two neighboring stationary points to determine which particular processes are mainly affected by changing the computational scheme from the PES scan to the umbrella sampling simulations. The results are summarized in Figure 5.

The first step is the nucleophilic attack of the catalytic water molecule on the phosphorus atom of the γ-phosphate group, accompanied by the cleavage of the bridging P^G^–O^3B^ bond between the γ- and β-phosphate groups. Therefore, the reaction coordinate is chosen as the difference of these two distances. This is important for the proper sampling because the reaction coordinate is mostly determined by the changes of the d(P^G^…O_W_) in the reactant region and by d(P^G^–O^3B^) close to the I1.

Figure 5 demonstrates that the energy barrier on the first step in the forward direction is much lower in the PES scan as compared with the QM/MM MD simulations. More precisely, the energy barrier obtained in this study from MD simulations is 11.1 kcal/mol and 3.1 kcal/mol on the potential energy surface. To clarify this issue, we carefully analyzed geometry parameters at this step (Figure 4, Table 1). First of all, the reaction coordinate, d(P^G^…O^3B^)–d(P^G^…O_W_), is 0.92 Å on the PES, whereas the umbrella integration gives 1.21 Å with the same functional. The reaction coordinate distribution in the unconstrained MD simulation of the ES complex is 1.18 ± 0.18 Å with the same DFT functional. If we mark this point on the Gibbs energy scan obtained in this study, we can estimate that the geometry configuration obtained on PES is approximately 1 kcal/mol higher in energy than the minimum obtained averaging different conformations from the umbrella sampling calculations. The TS1 position is also different. For the particular structure of PES, it is ~0.2 Å shifted to the negative values if averaging over a set of structures in the MD run. The transition state obtained on the PES is about 0.8 kcal/mol lower in energy than the TS1 obtained from the MD simulations. It should be noted that we consider only reaction coordinate values and do not evaluate contributions from other internal coordinates, for example, hydrogen bonds. We also keep in mind that the energy correction terms for the potential energy are calculated for the single point using the rigid rotor harmonic oscillator approximation.

The results of the present QM/MM MD calculations show that the entropic contribution directly evaluated from MD trajectories is the most essential only at the first reaction step, that is, the immediate nucleophilic attack of the water molecule. This is explained by the observation that the catalytic water molecule is labile and occupies a large number of conformations. In the following reaction steps, the energy differences between neighboring stationary points do not exceed 2 kcal/ mol, when calculated from the potential energy scans and in the QM/MM MD simulations (see Figure 5).

The reaction coordinate distributions in the unconstrained MD simulations are shown in Figure 4C. These are single-mode distributions for both functionals with similar mean values and standard deviations. If we consider the reaction coordinate of 0.92 Å, as in the ES minimum on PES, we observe that only 7.5% of the structures in the unconstrained MD simulations correspond to the reaction coordinates that are less than 0.92 Å. This indicates that the PES minimum structure is much tighter than the average MD structure; this explains a lower energy barrier in PES calculations.

The reaction coordinate differences are less pronounced in the I1 region. This is further evidence that the ES complex is rather flexible, but initiation of the reaction leads to the increase of the rigidity of the structure, thus minimizing the role of explicit conformational sampling.

The following two steps, I1 ⇌ I2 ⇌ I3^I^, are mostly related to the redistribution of protons as shown in Figure 1. The energy differences between the corresponding columns in Figure 5 do not exceed 2 kcal/mol, which is within the error bars of calculations with the DFT functionals [43]. The transitions between these minima occur with the low energy barriers; the I1, I2 and I3^I^ conformations have similar energy values that differ by less than 1.5 kcal/mol from the umbrella integration analysis. In the case of the PES scan, the I1 and I2 energies are the same, whereas the I3 is ~4 kcal/mol higher in energy than the ES. To further clarify this issue, we present the Markov state modeling results in the next subsection.

The rest of the profile refers to the taumerization of the Gln71 side chain from the imidic acid form to the amide form. The energy differences between two neighboring points do not exceed 2 kcal/mol (Figure 5), and the corresponding reaction coordinate values are similar (Table 1). Interestingly, the energy barrier corresponding to the I3^I^ → I4^I^ transition is smaller in the umbrella sampling simulations. This process corresponds to a rotation of the O^3G^Hε group around the P^G^–O^3G^ bond. Most likely, dynamical simulations allow one to find a set of similar pathways and some of them are slightly energetically more favorable due to local changes of the active site environment.

From these calculations, we formulate the following conclusions. First, explicit treatment of the protein dynamics is important at the first reaction step. This is due to the flexibility of the ES complexes, which is pronounced in the variations of the nucleophilic attack and P^G^–O^3B^ distances. Second, two different hybrid functionals, PBE0-D3 and ωB97x-D3, perform consistently in this application. Third, explicitly accounting for the entropic contributions using the umbrella sampling approach further decreases the effective rate constant to the value of 2·10^−4^ s^−1^ in the case of the PBE0 functional. This value is much lower than the experimentally observed 1.2 s^−1^; however, it is similar to the rate constant observed for the RP2-Glu138Gly mutant, 1.65·10^−4^ s^−1^ [21]. This prompted us to search for the alternative reaction pathway with the explicit role of RP2-Glu138, which is discussed in Section 3.4.

### 3.3. Markov State Model of the I1-I2-I3^I^ Transitions

**Figure 6:**
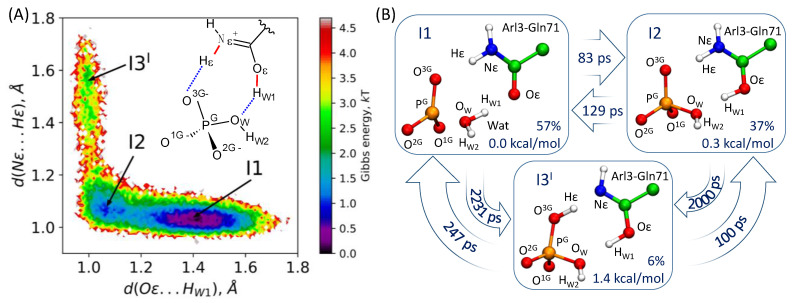
(**A**) Projection of the QM/MM MD data on the two interatomic distances d(H_W1_…Oε) and d(Hε…Nε); (**B**) Transitions between macrostates (I1, I2 and I3^I^) obtained in the MSM analysis. Relative populations of the corresponding macrostates and their relative energies are indicated in the boxes. Values in arrows report the transition times.

The MSM analysis was performed to delineate the energy landscape in the region of the unstable reaction intermediates I1 ⇌ I2 ⇌ I3^I^. The umbrella integration method for these elementary steps is characterized by the errors of 0.5 ÷ 1 kcal/mol, which are comparable with the energy differences between the I1, I2 and I3^I^ conformations. The energy barriers do not exceed 2 kcal/mol; therefore, MD simulations can efficiently sample this region. We calculated several QM/MM MD trajectories with a total length of 35 ps and performed the MSM analysis with respect to two coordinates—d(H_W1_…Oε) and d(Hε…Nε)—which are shown in Figure 1 and Figure 6, as described in the Methods section. We calculated the characteristic times of transitions between the I1, I2 and I3^I^ states and the corresponding rate constants (Figure 6). These data were utilized to estimate fractions of each intermediate and their energies relative to the I1. We compared these values with those obtained from the umbrella sampling simulations. The I2 energy relative to I1 is 0.3 kcal/mol in the MSM analysis and 0.1 kcal/mol in the umbrella sampling simulations. The I3^I^ energy is 1.4 kcal/mol in the MSM analysis and 1.8 kcal/mol in the umbrella sampling simulations. We conclude that the I1, I2 and I3^I^ intermediates occur in the pre-equilibrium state during the reaction, and the population of the I1 and I2 conformations are comparable, whereas the I3^I^ conformation is much less populated.

### 3.4. Enzyme Regeneration Pathways

**Figure 7:**
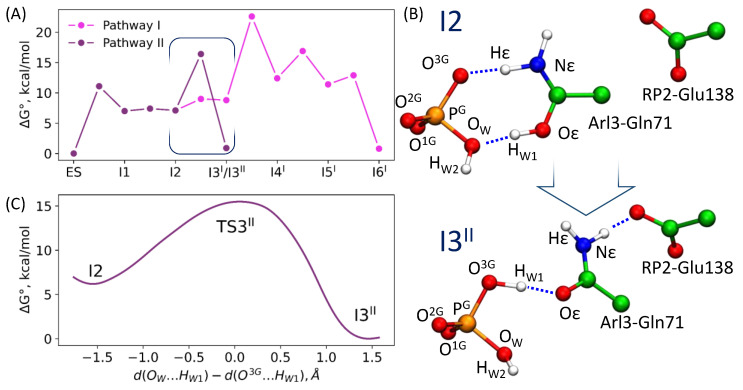
(**A**) Gibbs energy profiles of the reaction mechanisms recalculated by motifs of Ref. [26] (pathway I) and of the mechanism proposed in this study (pathway II); (**B**) The fragments of the active site corresponding to the I2 and I3^II^ conformations; (**C**) Gibbs energy profile of the I2 ⇌ I3^II^ transition.

So far, we have described the reaction route revealed in the previous QM/MM calculations and confirmed in the present QM/MM MD simulations. We call it pathway-I, and the reaction intermediates and transition states along this route are marked by the superscript “I”. Here, we describe an alternative route (pathway-II), revealed in the present QM/MM MD simulations. For this goal, we analyzed the experimental data on point mutations [21]. The RP2-Glu138 is located quite apart from the active site (Figure 2); however, its mutation results in the decrease of the reaction rate up to the values found for the intrinsic hydrolysis by Arl3 [21]. From the results presented in Section 3.2 and Section 3.3, we also learned that the I2 intermediate is ~6 times more populated than I3^I^ (Figure 6). This intermediate corresponds to the protonated form of the Arl3-Gln71 side chain. Therefore, we explored pathway-II, assuming the shift of Gln71 toward a prospective hydrogen-bond partner, the RP2-Glu138. This re-arrangement is coupled with proton transfer from Oε(Gln71) to the inorganic phosphate (Figure 7). To quantify this transition, we suggested the reaction coordinate for the umbrella sampling simulations as a difference between two distances, d(O_W_…H_W1_) and d(O^3G^…H_W1_). Calculations performed with the PBE0-D3 functional allowed us to estimate the energy barrier of transition from I2 to I3^II^ as 9.3 kcal/mol (the superscript “II” distinguishes the intermediates on pathway-II).

Pathway-II is comprised of three elementary steps, the first and second are the same as in the reaction mechanism proposed in Ref. [26]. The I2 intermediate corresponds to the stage at which the products of GTP hydrolysis, GDP and Pi (H_2_PO_4_^−^), are already formed. However, the side chain of Gln71 is in the protonated state. In pathway-I, four elementary steps were required to recover the amide form of the side chain of Gln71 with the relative energy of TS3^I^ being more than 20 kcal/mol higher in energy than that of the ES. The third step of pathway-II corresponds to a considerably lower energy barrier. The level of TS3^II^ is only 16.4 kcal/mol higher in energy than that of the ES. Thus, the effective rate constant of such reaction is 7 s^−1^, which is in perfect agreement with the experimental value of 1.2 s^−1^ [21].

## 4. Discussion

As mentioned in the Introduction, it is important to compare the minimum energy reaction profile computed with QM/MM approaches and the free-energy profile of the same reaction (here, for the Arl3-RP2 catalysis) computed by the QM/MM MD method using similar QM/MM procedures to construct interaction potentials. Analyzing the results, we find that many important features of the Arl3-RP2 reaction mechanism obtained by both approaches are the same. Previous QM/MM PES calculations [26] revealed that the reaction pathway could be subdivided into the steps of GTP decay and the steps of enzyme regeneration. Product species, GDP and Pi, are formed in the first part of the reaction pathway, but the side chain of the catalytically important Gln71 from Arl3 appears in the imide tautomer configuration. The second part of the reaction mechanism accounts for the restoration of the amide configuration of Gln71.

Newly performed calculations of this reaction mechanism, this time using QM/MM MD to construct the Gibbs energy profile, basically lead to the same qualitative conclusions. Moreover, the computed energy profile in the first reaction part shows the same reaction intermediates (I1, I2, I3^I^) as identified in QM/MM calculations, including the unstable intermediate I2 with the temporarily protonated glutamine side chain Gln71. The major difference in the profiles obtained with QM/MM and with QM/MM MD are related to the barrier height to the first reaction of intermediate I1. The potential energy difference between the TS1 and ES points on PES computed in [26] is about 4 kcal/mol, whereas the Gibbs energy difference is about 12 kcal/mol. Qualitatively, the fragment ES → TS1 → I1 of the reaction pathway is described similarly in both QM/MM and QM/MM MD simulations, in the sense that this is an elementary step of the P^G^-O^3B^ bond cleavage in GTP upon nucleophilic attack of the water molecule (Figure 4, Table 1). The structures of ES, TS1 and I1 as the stationary points in QM/MM and the structures recognized in the corresponding frames along MD trajectories are fairly close. We also point out that computational protocols (composition of the QM subsystem, theory level of quantum chemistry method, etc.) are very similar. Therefore, we can attribute the difference in the computed barrier heights (4 kcal/mol vs 12 kcal/mol) to the dynamical properties of ES complexes. The QM/MM minimization is most favorable for the reaction conformation, the energy of which is much closer to the energy of the first transition state than that of the non-reactive conformations.

In the reaction segment between I1 and I3^I^, both energy profiles look similar, demonstrating shallow reliefs with tiny energy barriers. It is consistent across QM/MM PES studies, QM/MM MD umbrella sampling simulations and MSM analysis of unconstrained QM/MM MD trajectories. The I2 state is considerably more populated than the I3^I^, thus prompting us to find another pathway starting from this intermediate.

The scenario of the reaction at the second segment, from I3^I^ to EP, as found in QM/MM calculations [26], was explored in the present QM/MM MD simulations. Figure 5 shows that the computed Gibbs energy profile is basically consistent with the QM/MM profile on PES. No notable differences between the results of the two functionals are detected.

However, the present QM/MM MD calculations reveal another mechanism of enzyme regeneration as compared to the previously proposed pathway-I. In QM/MM calculations [26], rotations around the P-O bonds in Pi and proton transfer were considered to compute minimum energy routes to arrive at EP. A barrier of about 15 kcal/mol between the I3^I^ and I4^I^ on PES was estimated. A related set of reaction coordinates was tested in QM/MM MD calculations (Table 1), producing a similar energy profile (Figure 5).

The analysis of QM/MM MD trajectories in the vicinity of the I2 structure prompted us to explore another reaction route, pathway-II, which we described in the Results section. The rate constant k_cat_, evaluated with the transition state theory with pathway-II, 7 s^−1^, is consistent with the experimental data (1.2 s^−1^); however, this does not reject the occurrence of pathway-I. Moreover, we note the important role of the residue Glu138 from RP2, when pathway-II is considered. As recognized in previous experiments [21], the mutation of Glu138 to Gly drastically reduces the rate of GTP hydrolysis. As such, the scenario corresponding to pathway-I can be an option for the Glu138Gly mutant of RP2.

It should be noted that the malfunction of Arl3-RP2 machinery due to point mutations may severely affect human vision [20]. Interestingly, physiologically important mutations in the Arl3-RP2 complex are in the accelerating protein RP2 [21,22] but not in the GTPase itself, as in many GTPases. According to [21], the key patient mutations in RP2, Arg118Ala and Glu138Gly abolish the GTP hydrolysis acceleration, resulting in several eye diseases including hereditary blindness. The arginine finger Arg118 from RP2 properly positions the Gln71 side chain. Moreover, according to the simulations, Gln71 actively contributes to the reaction mechanism. The experiments [21] also show that the Gln71Leu mutation in Arl3 is hydrolysis-deficient, demonstrating the importance of Gln71 in the reaction.

The glutamine residue at this position in the enzyme active site is common for many GTPases, in particular, for Gln61 from GTPase Ras in the widely studied complex Ras-GAP (p21Ras-p120GAP). During the long history of studies of the GTP hydrolysis mechanism in GTPases [18,44,45,46,47,48], several proposals have been put forward concerning the role of this residue for the GTPase catalysis. There is a consensus about the importance of this residue for positioning the nucleophile water molecule for an attack on the γ-phosphate; however, a more active role of Gln61 in the Ras-GAP catalyzed GTP hydrolysis is still debated [30,31,32]. In line with the previous simulations of GTP hydrolysis by Ras-GAP [30] consistent with the glutamine-assisted mechanism, pathway-I of this reaction in Arl3-RP2 assumes that the side chain of Gln71 participates in proton transfer after cleavage of the of the O^3B^–P^G^ bond in GTP. As a result, the protonated form of Gln71 appears in the intermediate I2, and the imide tautomer of the glutamine side chain appears in the intermediate I3^I^. Pathway-II does not suggest the appearance of the imide form of Gln71, but actively explores the occurrence of the intermediate I2 with the protonated form of Gln71.

## 5. Conclusions

The described QM/MM MD calculations of the Gibbs energy profiles of the GTP hydrolysis chemical reaction catalyzed by the Arl3-RP2 protein complex allowed us to formulate two possible reaction pathways. Pathway-I reproduces the features of the mechanism formulated previously, on the basis of the minimum energy profile on the potential energy surface obtained in the QM/MM calculations. Pathway-II, which is consistent with the experimental kinetic studies of the wild-type protein, assumes a different scheme of enzyme regeneration than pathway-I. Both schemes agree that the role of Gln71 from Arl3 is not restricted by the positioning of the nucleophilic water molecule in the enzyme–substrate configuration, but the glutamine side chain actively participates in the reaction. This is in line with the experimental observation that the mutation of Gln71 by leucine abolishes the hydrolysis. The results obtained from the molecular simulations extend the knowledge on the mechanism of GTP hydrolysis reaction in small GTPases. From the methodology point of view, the results of the present QM/MM MD calculations show that the entropic contribution directly evaluated from MD trajectories significantly changes the Gibbs energy profile only at the first reaction step, that is, the immediate nucleophilic attack of the water molecule occurs. This is explained by the observation that the catalytic water molecule is labile and occupies a large number of conformations. We also demonstrate that the utilization of QM/MM MD protocols with different hybrid functionals (PBE0-D3 and ωB97x-D3) leads to consistent results.

## Figures and Tables

**Table 1:** Reaction coordinates utilized in the umbrella sampling of the Gibbs energy surface. Reaction coordinate values are calculated for stationary points in MD simulations with the ωB97x-D3 and PBE0-D3 functionals in this study and with the PBE0-D3 in [26] are also shown.

Reaction Coordinate	Step	Stationary Point	Reaction Coordinates at Stationary Points, Å			
			MD Simulations		PES Scan	
			ωB97x-D3	PBE0-D3		PBE0-D3
d(P^G^…O^3B^)–d(P^G^…O_W_)	1	ES	1.25	1.21		0.92
		TS1	−0.19	−0.21		0.02
		I1	−1.09	−1.09		−0.93
d(Oε…H_1w_)–d(Hε…Nε)	2, 3^I^	I1	0.45	0.33		0.30
		TS2	0.13	0.15		0.16
		I2	−0.02	0.00		−0.04
		TS3^I^	−0.28	−0.29		−0.32
		I3^I^	−0.60	−0.49		−0.43
d(O^3B^…Hε)	4^I^	I3^I^	3.81	3.77		3.86
		TS4^I^	2.60	2.58		2.81
		I4^I^	1.40	1.42		1.41
d(H_2w_…Nε)	5^I^	I4^I^	4.10	3.98		3.80
		TS5^I^	2.86	2.79		2.31
		I5^I^	1.69	1.55		1.54
d(H_1w_…O_w_)	6^I^	I5^I^	2.40	2.54		1.73
		TS6^I^	1.88	1.94		1.63
		I6^I^	0.99	1.00		1.02

## Data Availability

Coordinates of model systems at the stationary points regions are deposited to the general-purpose open-access repository ZENODO (https://doi.org/10.5281/zenodo.5033544, accessed on 29 June 2021).

## Sample Availability

Samples of the compounds are not available from the authors.
